# New insights into the genetic diversity of *Leishmania RNA Virus 1* and its species-specific relationship with *Leishmania* parasites

**DOI:** 10.1371/journal.pone.0198727

**Published:** 2018-06-18

**Authors:** Lilian Motta Cantanhêde, Flavia Gonçalves Fernandes, Gabriel Eduardo Melim Ferreira, Renato Porrozzi, Ricardo de Godoi Mattos Ferreira, Elisa Cupolillo

**Affiliations:** 1 Laboratório de Epidemiologia Genética, Fundação Oswaldo Cruz, Unidade Rondônia, Porto Velho, Rondônia, Brazil; 2 Laboratório de Pesquisa em Leishmaniose, Instituto Oswaldo Cruz, Fundação Oswaldo Cruz, Rio de Janeiro, Rio de Janeiro, Brazil; Bernhard Nocht Institute for Tropical Medicine, Hamburg, Germany, GERMANY

## Abstract

Cutaneous leishmaniasis is a neglected parasitic disease that manifests in infected individuals under different phenotypes, with a range of factors contributing to its broad clinical spectrum. One factor, *Leishmania RNA Virus* 1 (LRV1), has been described as an endosymbiont present in different species of *Leishmania*. LRV1 significantly worsens the lesion, exacerbating the immune response in both experimentally infected animals and infected individuals. Little is known about the composition and genetic diversity of these viruses. Here, we investigated the relationship between the genetic composition of LRV1 detected in strains of *Leishmania* (*Viannia*) *braziliensis* and *L*. (*V*.) *guyanensis* and the interaction between the endosymbiont and the parasitic species, analyzing an approximately 850 base pair region of the viral genome. We also included one LRV1 sequence detected in *L*. (*V*.) *shawi*, representing the first report of LRV1 in a species other than *L*. *braziliensis* and *L*. *guyanensis*. The results illustrate the genetic diversity of the LRV1 strains analyzed here, with smaller divergences detected among viral sequences from the same parasite species. Phylogenetic analyses showed that the LRV1 sequences are grouped according to the parasite species and possibly according to the population of the parasite in which the virus was detected, corroborating the hypothesis of joint evolution of the viruses with the speciation of *Leishmania* parasites.

## Introduction

Tegumentary Leishmaniasis (TL) is a particularly problematic and neglected parasitic disease that is endemic in the South American tropics, in which parasites have the ability to occasionally migrate from the initial site of inoculation in a process called "infectious metastasis" [1], generating distinct phenotypes after infection.

Protozoans of the genus *Leishmania* are present in several regions of the world, causing approximately 12 million symptomatic cases. It is estimated that there are at least 120 million asymptomatic cases and approximately 1.7 billion people at risk of contracting the infection [2,3]. Contributing to this context of neglect are the complex epidemiology, ecology, lack of management tools, and scarcity of data [3].

*Leishmania* species involved in human infection are mostly (80%) dermotropic, infesting and ulcerating the human skin around the site of inoculation of their hematophagous vector. Several of the previously described parasite species are able to migrate to regions of the skin unrelated to inoculation, causing secondary lesions that often become severely inflamed, deforming the surrounding tissues [4]. The disease phenotypes are mainly distinguished by the infecting species. However, it is not fully understood which factors affect the disease’s severity and presentation [5]. The disease process is multifactorial, depending on complex interactions among parasites, hosts and environmental factors, including genetic and non-genetic factors [6]. In addition, a virus present in the cytoplasm of the *Leishmania* parasite, *Leishmania RNA virus* 1 (LRV1), has recently been shown to influence the development of mucosal lesions and the overall course of the disease [4,7].

The involvement of LRV1 in the evolution of leishmaniasis has mainly been observed in murine models. LRV1-bearing parasites repeatedly metastasized while LRV1-negative parasites did not, confirming the role of the virus in the severity of the disease [7,8]. In addition, the virus has been associated with mucosal leishmaniasis in humans [9], increasing the risk of therapeutic failure in patients infected by *L*. *braziliensis* [10] and the risk of first-line treatment failure and symptomatic relapse in patients infected with *L*. *guyanensis* [11].

*Leishmania* RNA viruses are classified in the *Totiviridae* family [12–14]. These viruses are composed of a double stranded RNA (dsRNA) genome of approximately 5.3 kb [15] that encodes a viral capsid protein and RNA-dependent RNA polymerase (RDRP) [14,16]. LRV1 was first molecularly described in a strain of *L*. *guyanensis* [15], and since then, the virus has been reported in several species of the parasite. LRV1 is often found in species of the subgenus *Viannia* scattered throughout the American continent and is found sporadically in Old World *Leishmania* species of the subgenus *Leishmania* (classified as LRV2) [16,17].

The presence of LRV1 was demonstrated in strains of the species *L*. *guyanensis* and *L*. *braziliensis* [9,11,18,19]. Current reports show that the infectious metastasis of *L*. *braziliensis* is not exclusively associated with the presence of LRV [9,20]. LRV1-positive isolates are still reported as having no association with particular manifestations in individual patients infected with *Leishmania* (*L*.) *amazonensis*, *Leishmania* (*V*.) *lainsoni* [9] or *Leishmania* (*L*.) *infantum* [21]. Recent epidemiological evidence suggests that the prevalence of LRV1 is highly variable among *Leishmania* species and that *Leishmania-*parasitized LRV1 only occur in some geographic regions [9,11,20,22–24].

The virus seems to have been present in parasites of the genus *Leishmania* from the time that vertebrate hosts became involved in the Leishmaniinae life cycle [25], but prior to the spread of these parasites around the world. According to phylogenetic studies, the genetic distance between the parasite species is similar to the distance between the virus species LRV1 and LRV2. In addition, genetic similarity is observed based on the geographic distribution of *Leishmania* spp. [16,26]. A recent study reported for the first time that level of genomic diversity among LRV1 in parasites of the *L*. *guyanensis* species is low and suggested evolutionary relationships between the viruses and the parasites [23].

Considering the importance of LRV1 in *Leishmania* parasites, our study aimed to analyze the LRV1 sequences in strains of different *Leishmania* species from the Brazilian Amazon region and compare those with LRV1 sequences available in public databases to contribute evidence on the hypothesis of the co-evolution of LRV1 and *Leishmania* (*Viannia*) parasites.

## Methodology

**Fig 1:**
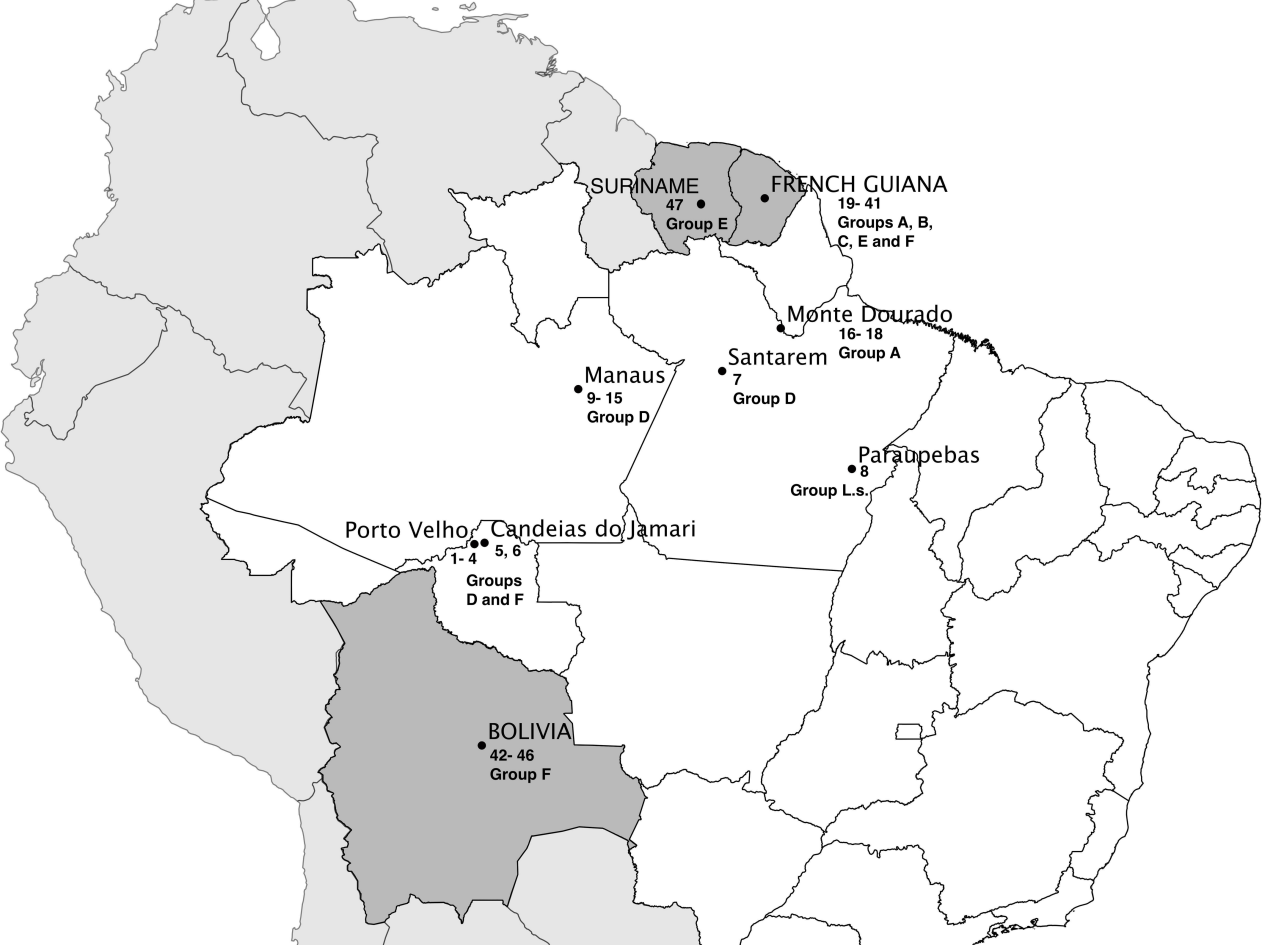
Partial map of South America indicating the geographic distribution of *Leishmania* strains carrying LRV1 sequences included in the study. Partial map of Brazil (white), divided by states. Neighboring countries are colored gray, with Bolivia, Suriname and French Guiana in dark gray. The groups are the same as those reported by Tirera et. al., 2017. Numbers indicate the sequences analyzed from each geographic region, following the ID numbers presented in Table 1. The map was constructed using Quantum GIS version 2.18.13 (http://download.qgis.org), the base layers were downloaded from Carlos Efrain Porto Tapiquen, Orogenesis Soluciones Geograficas, Porlamar, Venezuela, 2015, based on shapes from Environmental Systems Research Institute (ESRI) with free distribution (http://tapiquen-sig.jimdo.com). The points corresponding to cities and countries were obtained from Google Earth (http://earth.google.com).

47 LRV1 sequences were analyzed in this study. Of these, 34 are LRV1 sequences from *L*. *braziliensis* and *L*. *guyanensis* available in GenBank, corresponding to sequences reported from studies in French Guiana [23], Bolivia [5,27], and Brazil [5,27]. The LRV1 sequences obtained in the present study (n = 13) were from strains of *L*. *braziliensis*, *L*. *guyanensis* and *L*. *shawi* that were previously identified as LRV1 positive [9]. The sequences obtained were deposited in GenBank under accession numbers MG202139 through MG203151. The analyzed sequences correspond to LRV1 detected in strains of *Leishmania* isolated in different areas of the Amazon region, including areas of Brazil, Bolivia and French Guiana (Fig 1). All information on the LRV1 sequences analyzed in this study is presented in Table 1.

**Table 1:** Sequences used for phylogenetic and genetic divergence analyses.

ID	*Leishmania* Strain ID	Accession Number	*Leishmania* International code	Parasite species	Geographic Origin	Sequence length^1^	Nucleotide position (sequence length analyzed)^2^	Ref
1	3562*	MG202146	MHOM/BR/2014/308	*L*. *braziliensis*	Porto Velho/RO/Brazil	790	47–834 (788)	This study
2	386	MG202144	MHOM/BR/2015/386	*L*. *braziliensis*	Porto Velho/RO/Brazil	788	47–834 (788)	This study
3	3545*	MG202143	MHOM/BR/2014/275	*L*. *braziliensis*	Porto Velho/RO/Brazil	788	47–834 (788)	This study
4	3569*	MG202145	MHOM/BR/2014/299	*L*. *braziliensis*	Porto Velho/RO/Brazil	788	47–834 (788)	This study
5	3567*	MG202139	MHOM/BR/2014/291	*L*. *braziliensis*	Candeias/RO/Brazil	788	47–834 (788)	This study
6	3538*	MG202140	MHOM/BR/2014/271	*L*. *guyanensis*	Candeias/RO/Brazil	788	47–834 (788)	This study
7	3354*	MG202141	MHOM/BR/2011/S77-ABF	*L*. *guyanensis*	Santarem/PA/Brazil	787	47–834 (788)	This study
8	1545*	MG202142	MCEB/BR/1984/M8408	*L*. *shawi*	Paraupebas/PA/Brazil	788	47–834 (788)	This study
9	3460*	MG202147	MHOM/BR/2013/04LTAVMR	*L*. *guyanensis*	Manaus/AM/Brazil	788	47–834 (788)	This study
10	3461*	MG202148	MHOM/BR/2013/05LTAMVL	*L*. *guyanensis*	Manaus/AM/Brazil	788	47–834 (788)	This study
11	3486*	MG202149	MHOM/BR/2013/27JNS	*L*. *guyanensis*	Manaus/AM/Brazil	788	47–834 (788)	This study
12	3542*	MG202151	MHOM/BR/2014/233CFS	*L*. *guyanensis*	Manaus/AM/Brazil	737	47–834 (788)	This study
13	3503*	MG202150	MHOM/BR/2013/33LGS	*L*. *guyanensis*	Manaus/AM/Brazil	788	47–834 (788)	This study
14	1398*	JX313127	MHOM/BR/1989/IM3597	*L*. *guyanensis*	Manaus/AM/Brazil	3228	370–3616 (3247)	[19]
15	2001	KY750607	MHOM/FG/2011/2001	*L*. *guyanensis*	Manaus/AM/Brazil**	5196	62–5278 (5217)	[23]
16	M4147	KX808487	MHOM/BR/1975/M4147	*L*. *guyanensis*	Monte Dourado/PA/Brazil	5283	1–5307 (5307)	[5]
17	M4147	U01899	MHOM/BR/1975/M4147	*L*. *guyanensis*	Monte Dourado/PA/Brazil	5283	371–834 (464)	[28]
18	M5313	JX313126	IWHI/BR/1978/M5313	*L*. *guyanensis*	Monte Dourado/PA/Brazil	2480	371–834 (464)	[19]
19	LF94	KY750608	MHOM/FG/2013/LF94	*L*. *guyanensis*	French Guiana	5192	62–5278 (5217)	[23]
20	XJ93_2	KY750609	MHOM/FG/2013XJ93	*L*. *guyanensis*	French Guiana	5195	62–5278 (5217)	[23]
21	YA70	KY750610	MHOM/FG/2013YA70	*L*. *braziliensis*	French Guiana	5188	62–5278 (5217)	[23]
22	2014	KY750611	MHOM/FG/2013/2014	*L*. *guyanensis*	French Guiana	5193	62–5278 (5217)	[23]
23	2008	KY750612	MHOM/FG/2012/2008	*L*. *guyanensis*	French Guiana	5194	62–5278 (5217)	[23]
24	2015	KY750613	MHOM/FG/2012/2015	*L*. *guyanensis*	French Guiana	5194	62–5278 (5217)	[23]
25	2028_1	KY750614	MHOM/FG/20122028	*L*. *guyanensis*	French Guiana	5194	62–5278 (5217)	[23]
26	2028_2	KY750615	MHOM/FG/2012/2028	*L*. *guyanensis*	French Guiana	5194	62–5278 (5217)	[23]
27	2028_3	KY750616	MHOM/FG/2012/2028	*L*. *guyanensis*	French Guiana	5194	62–5278 (5217)	[23]
28	LF98	KY750617	MHOM/FG/2013/LF98	*L*. *guyanensis*	French Guiana	5194	62–5278 (5217)	[23]
29	LL28	KY750618	MHOM/FG/2012/LL28	*L*. *guyanensis*	French Guiana	5194	62–5278 (5217)	[23]
30	MC71	KY750619	MHOM/FG/2012/MC71	*L*. *guyanensis*	French Guiana	5194	62–5278 (5217)	[23]
31	MJ25	KY750620	MHOM/FG/2012/MJ25	*L*. *guyanensis*	French Guiana	5194	62–5278 (5217)	[23]
32	PD46	KY750621	MHOM/FG/2014/PD46	*L*. *guyanensis*	French Guiana	5194	62–5278 (5217)	[23]
33	VL91	KY750622	MHOM/FG/2012/VL19	*L*. *guyanensis*	French Guiana	5194	62–5278 (5217)	[23]
34	VW21	KY750623	MHOM/FG/2013/VW21	*L*. *guyanensis*	French Guiana	5194	62–5278 (5217)	[23]
35	WF69_G1_	KY750624	MHOM/FG/2012/WF69	*L*. *guyanensis*	French Guiana	5194	62–5278 (5217)	[23]
36	WF69_G2_	KY750625	MHOM/FG/2012/WF69	*L*. *guyanensis*	French Guiana	5194	62–5278 (5217)	[23]
37	XJ93_G1_	KY750626	MHOM/FG2013/XJ93	*L*. *guyanensis*	French Guiana	5194	62–5278 (5217)	[23]
38	XK73	KY750627	MHOM/FG/2013/XK73	*L*. *guyanensis*	French Guiana	5194	62–5278 (5217)	[23]
39	YE48	KY750628	MHOM/FG/2013/YE48	*L*. *guyanensis*	French Guiana	5194	62–5278 (5217)	[23]
40	YR07	KY750629	MHOM/FG/2014/YR07	*L*. *guyanensis*	French Guiana	5194	62–5278 (5217)	[23]
41	YZ58	KY750630	MHOM/FG/2012/YZ58	*L*. *guyanensis*	French Guiana	5194	62–5278 (5217)	[23]
42	LEM2700	KX808483	MHOM/BO/1990/AN	*L*. *braziliensis*	Bolivia	5283	3–5308 (5306)	[5]
43	LEM3874	KX808486	MHOM/BO/IMT252	*L*. *braziliensis*	Bolivia	5286	3–5311 (5309)	[5]
44	LEM2780(b)	KX808485	MHOM/BO/1990/CS	*L*. *braziliensis*	Bolivia	5274	18–5299 (5281)	[5]
45	LEM2780(a)	KX808484	MHOM/BO/1990/CS	*L*. *braziliensis*	Bolivia	5259	1–5297 (5297)	[5]
46	Lb2169	KC862308	MHOM/BO/2011/2169	*L*. *braziliensis*	Bolivia	4969	371–834 (464)	[27]
47	CUMC1	M92355	MHOM/SR/1980/CUMC1	*L*. *guyanensis*	Suriname	5284	2–5313 (5312)	[27]

*IOC/L code related to strains deposited in the *Leishmania* Collection from the Oswaldo Cruz Foundation

** isolated in French Guiana, but the geographic location of infection was reported as in the region of Manaus, Amazonas state (AM), Brazil [23]

^1^Total length of the sequence available in the Genbank or obtained in this study

^2^Nucleotide position and sequence length defined after sequences alignment.

MHOM = Mammalia, *Homo sapiens*; MCEB = Mammalia, *Cebus apella*; IWHI = *Insecta*, *Lutzomyia whitmani*; *L*. *guyanensis* = *Leishmania* (*Viannia*) *guyanensis*; *L*. *braziliensis* = *Leishmania* (*Viannia*) *braziliensis*; *L*. *shawi* = *Leishmania* (*Viannia*) *shawi*. AM, PA and RO are Amazonas, Pará and Rondônia states, respectively.

### *Leishmania* culture, RNA extraction, cDNA synthesis and sequencing

The strains of *Leishmania* used in this study were grown in NNN (Novy-MacNeal-Nicolle) and Schneider (Sigma®, St. Louis, MO, USA) biphasic culture medium supplemented with 30% fetal bovine serum (Vitrocell®, Campinas, SP, BR) and 2% filtered human urine and were subsequently incubated in a BOD (biochemical oxygen demand) incubator. After an average of 7 days of incubation, 10 mL of 1x10^5^ parasites was centrifuged at 5000 *g* for 5 minutes to obtain the parasite’s mass.

RNA was extracted using a Purelink Genomic RNA Mini Kit (Ambion®, Carlsbad, CA, USA) following the protocol proposed by the manufacturer from 300 μL of culture medium containing 1x10^6^ parasites per mL. The concentration of total extracted RNA was analyzed in a NanoDrop™ spectrophotometer (Thermo Scientific Fisher, Waltham, MA, USA).

A SuperScript ™ III First-Strand Synthesis System (Invitrogen, Carlsbad, CA, USA) was used to synthesize cDNA from 150 ng of total RNA with random primers, performed according to the manufacturer's protocol.

For primer design, the genomic sequences available in the GenBank database of the species LRV1-1 (NC002063) and LRV1-4 (NC003601) were retrieved, and after alignment with ClustalW2, the region with the greatest similarity between the two sequences was chosen using Primer-BLAST [29]. The primers LRV1 F *orf*1 5’-ATGCCTAAGAGTTTGGATTCG-3’ and LRV R *orf*2 5’-AATCAATTTTCCCAGTCATGC-3’ (sense primer located at positions 16 to 36 and antisense primer from 847 to 867, considering the genomes employed for primer design) were used, amplifying a fragment of approximately 850 base pairs corresponding to part of the *orf*1 region and the beginning of the *orf*2 region, including the portion responsible for encoding the viral capsid protein.

PCR reactions were performed with a final volume of 50 μL: 3 μL of cDNA, 0.4 μM of each primer, 0.3 μM of dNTP mix, 1x Buffer + MgCl_2_ and 1 U Taq DNA polymerase (Invitrogen, Life Technologies, Carlsbad, CA, USA). PCR was performed at 94°C for 2 minutes, followed by 30 cycles at 94°C for 30 seconds, 56°C for 45 seconds, and 72°C for 30 seconds, with a final extension phase at 72°C for 3 min. After the end of the cycle, the amplified fragments were visualized on a 1% Agarose gel stained with GelRed™ (Biotium, Hayward, CA, USA).

For sequencing, 45 μL of the PCR products were purified using the Purelink Quick Gel Extraction Kit (Invitrogen™, Carlsbad, CA, USA) following the manufacturer's recommendations, with a final elution volume of 30 μL. Sequencing reactions were performed with the same primers for PCR amplification, on an ABI3730 DNA analyzer using the ABI PRISM BigDyeTerminator v3.1Cycle Sequencing Kit at the Genomic Platforms from Fiocruz (Rio de Janeiro—RPT01A and Gonçalo Muniz Institute, Fiocruz Bahia -RPT01B). Both strands of each PCR product were sequenced. The quality of the DNA sequences was checked and overlapping fragments were assembled using the Phred/Phrap/Consed package [30–32].

### Analyses of LRV1 sequences

The sequences obtained in this study were visualized and edited in the program BioEdit [33]. All sequences were aligned and manually verified using the software MEGA7 [34].

Two datasets were analyzed: (i) all 47 LRV1 sequences of different size (Table 1), allowing for the analysis of a total of 351 positions in the final dataset, named dataset I; and (ii) 43 LRV1 sequences, excluding those with only a few positions in common with the bulk of the sequences obtained in the present study (M5313, 1398, 3542, and 2169), resulting in a total of 687 positions in the final dataset, named dataset II. All positions containing gaps and missing data were eliminated. The programs MEGA7 [34] and SplitsTree 4.14.6 [35] were used to determine the sequence characteristics.

To infer the best model for phylogenetic tree construction, the “find the best DNA model” option available in MEGA7 was used, and the model with the lowest BIC (Bayesian Information Criterion) score was considered to be the one that best described the substitution pattern in the analyzed sequences. The 1992 Tamura distance method (T92) [36] with gamma distribution shape parameter (G) and invariable sites (I) was employed to evaluate the genetic distance between the sequences, and the 10,000 replicate (bootstrap) maximum likelihood method was used to construct the tree in MEGA7 software [34]. The sequences were also compared with each other to estimate the level of similarities between them.

In addition, to increase the chances of finding the most parsimonious connections, networks were built using the SplitsTree program. The phylogenetic networks were constructed using NeighborNet and a minimum spanning network.

## Results

### Characteristics of the sequences

The sequences ranged from 737 to 5286 nucleotides in length (Table 1). In the first dataset (complete dataset of 47 sequences), a total of 2852 sites were conserved, with 2444 variable and 2157 parsimonious informative (PI) sites, of which 238 (11%) were detected in the region corresponding to the sequences obtained in the present study. In the second dataset [43 sequences (excluding 1398, M5313, 2129, and 3542), 788 nucleotides in length], a total of 458 sites were conserved, with 315 variable sites and 239 PI sites, corresponding to 30.33% of the analyzed region.

The nucleotide composition is 26.3% (U), 21.3% (C), 27.9% (A), and 24.5% (G). A Phi test was conducted using SplitsTree, and no statistically significant evidence of recombination (p = 0.99) was observed [37].

### Cluster definition and sequence similarities

**Fig 2:**
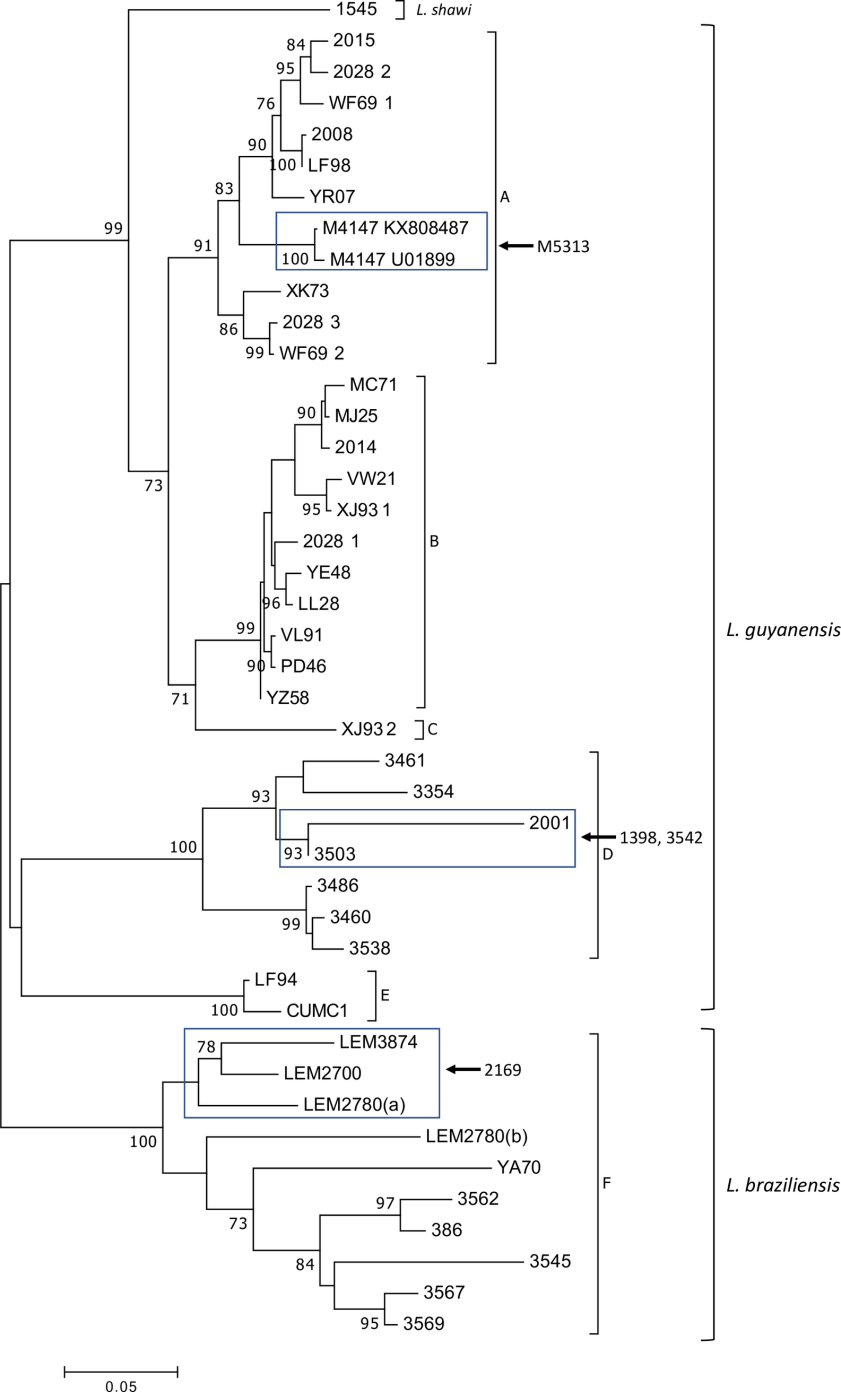
Maximum likelihood tree of *Leishmania* RNA Virus 1 detected in *Leishmania* (*Viannia*) species from South American countries. The analysis involved 43 nucleotide sequences. There was a total of 687 positions in the final dataset (Dataset I). Boxes indicate the clustering of M5313, 1398, 3542, and 2169 after analysis involving 47 nucleotide sequences, with a total of 351 positions. Groups A-F were defined as previously reported [23]. The tree was inferred based on the Tamura 92 model with a Gamma distribution and invariable sites (I). Bootstrap values (after 10,000 replicates) above 70% are shown. Sites containing gaps and missing data were excluded from the analysis. L.b. = *L*. *braziliensis*; L.g. = *L*. *guyanensis*; L.s. = *L*. *shawi*. For details of samples see Table 1.

Independently of the dataset analyzed, the six clusters defined by Tirera et al. in 2017 using complete viral genome sequences were observed in the present study, and all groups were well supported in the maximum likelihood tree (Fig 2), although only a small region of the viral genome was considered. Few differences were observed among the trees obtained for each dataset, mainly related to the bootstrap value, but here we only considered the values above 70%, and this did not change among the analyses (S1 Fig).

**Fig 3:**
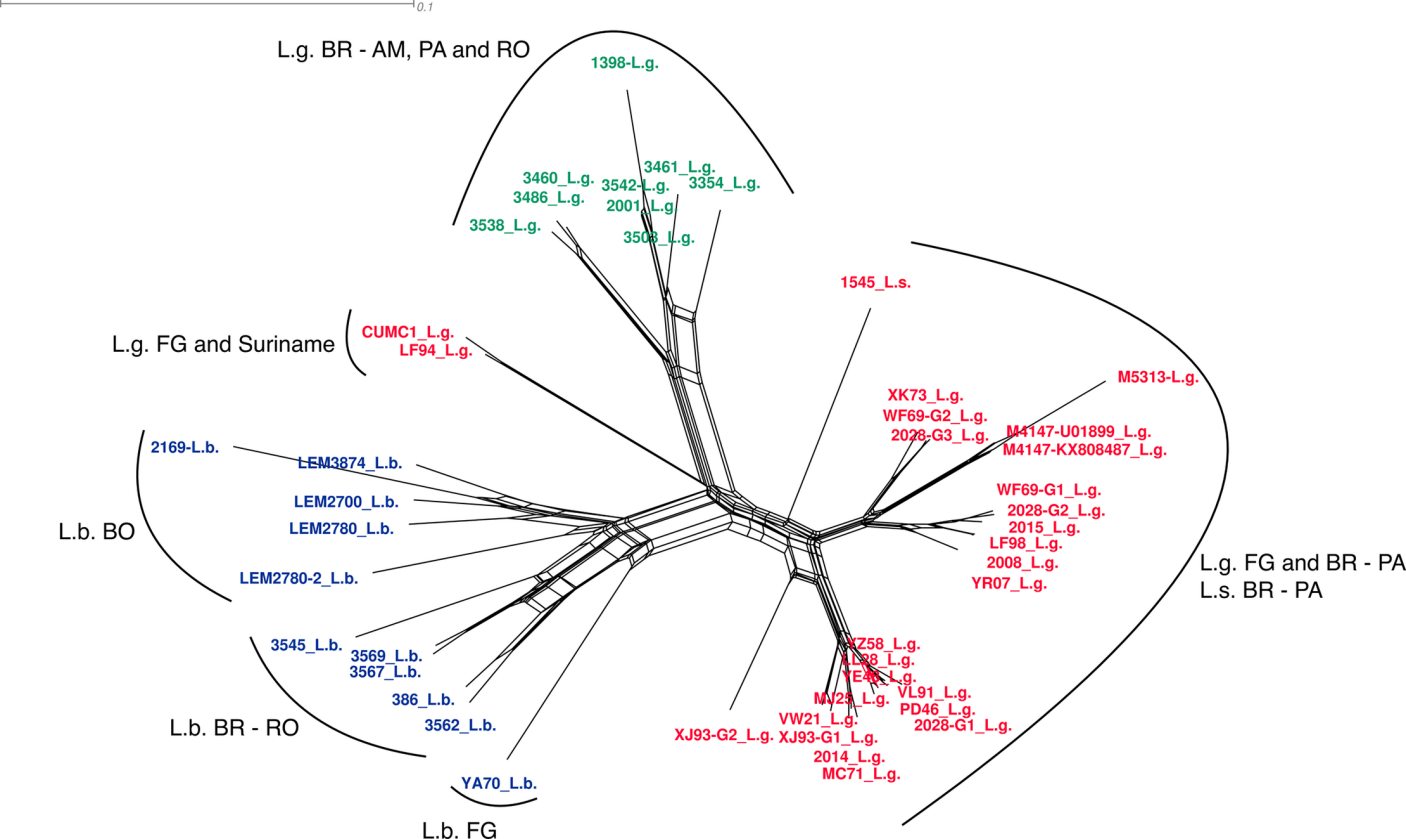
NeighborNet presenting the relationship among LRV1 sequences from different *Leishmania* (*Viannia*) species. The network was computed using SplitsTree software. EqualAngle was employed for splits transformation. Text colors refer to groups from Fig 4: red = cluster I, blue = cluster II, and green = cluster III. For sample details see Table 1.

The groups observed on the maximum likelihood tree are also evident in NeighborNet (Fig 3). Considering the relationship between the groups observed in the present study and those from Tirera et al 2017, we used the same description to present the observed clusters. All *L*. *guyanensis* LRV1 sequences obtained in the present study were placed in Group D, which, in the previous study, was represented by an LRV1 sequence from *L*. *guyanensis* isolated in Brazil (1398—JX313127) and an LRV1 sequence from *L*. *guyanensis* (2001- KY750607) isolated from a patient who acquired the infection in Brazil (Manaus, Amazonas), as reported by Tirera et al. 2017. The sequences not included in the maximum likelihood tree (Fig 2) integrated the NeighborNet (Fig 3) and clustered as indicated in the maximum likelihood analysis when less characters were analyzed: *L*. *guyanensis* LRV1 M5313 close to both M4147 sequence, 1398 and 3542 with the others sequences from Group D, and 2169 in the *L*. *braziliensis* LRV1 Group F.

**Fig 4:**
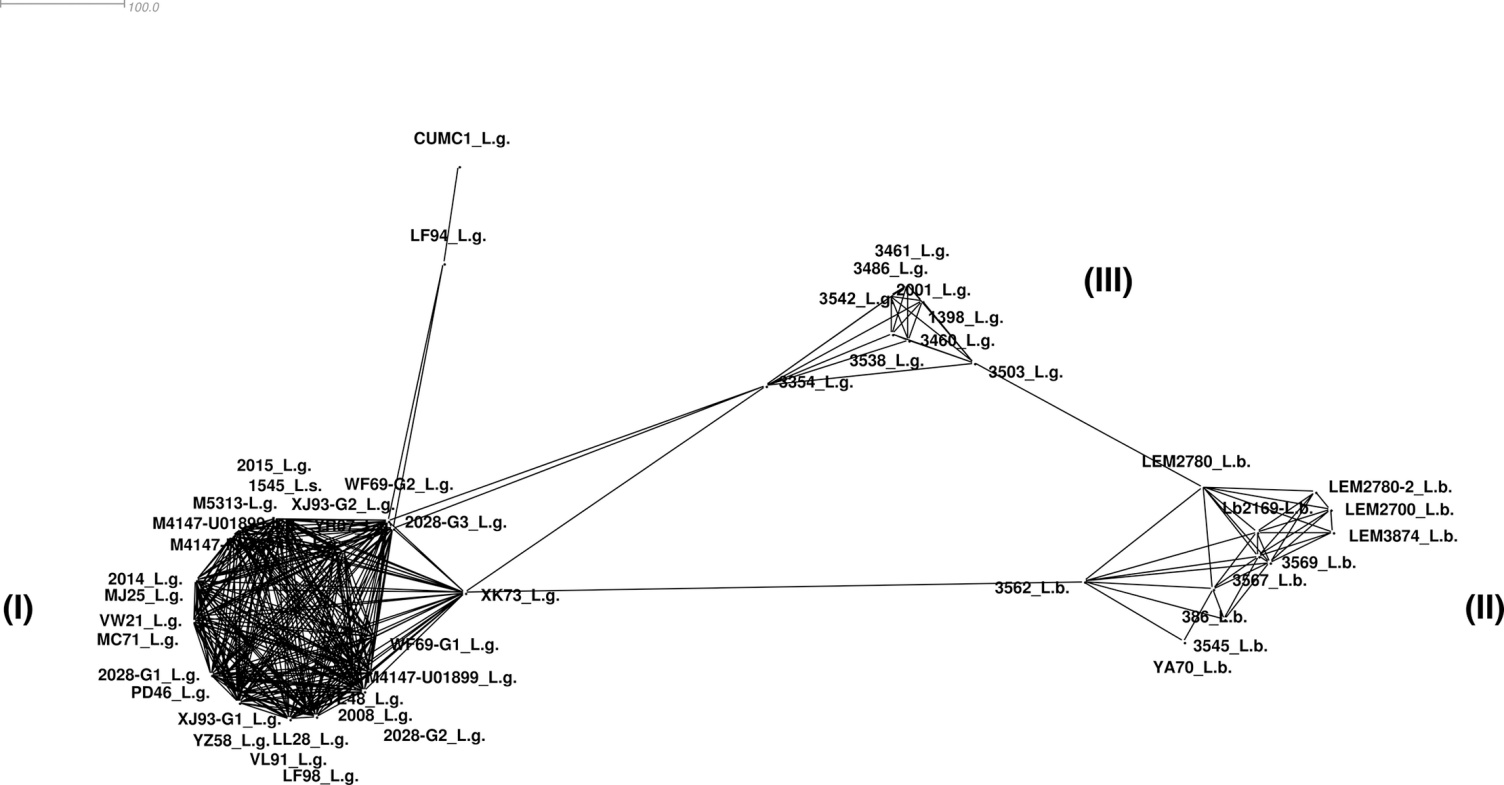
Minimum spanning network displaying the connections among LRV1 sequences from different *Leishmania* (*Viannia*) species. I = LRV1 sequences from *L*. *guyanensis* groups A, B, C and E, and *L*. *shawi*; II = LRV1 sequences from *L*. *braziliensis* group; III = LRV1 sequences from *L*. *guyanensis* group D. For sample details, see Fig 1 and Table 1.

Groups A through E represent sequences of the *L*. *guyanensis* virus, and Group F contains LRV1 sequences from *L*. *braziliensis*. In addition, for the first time, we analyzed the sequence of an LRV1 detected in *L*. *shawi* (1545 –MG202142), which was identified as L.s. in the ML tree, NeighborNet, and the MinSpanning Network (Figs 2–4).

Three clusters were present in the MinSpanning Network, one composed of LRV1 sequences from groups A, B, C and E from *L*. *guyanensis* (I) as well as the LRV1 sequence from *L*. *shawi*; the second composed of *L*. *braziliensis* alone (II), and the third composed of only *L*. *guyanensis* LRV1 sequences from Group D (III) (Fig 4).

A high degree of similarity was observed among the sequences, ranging from 79.6% to 99.9%. Considering LRV1 from the *L*. *guyanensis* strains, Group A exhibited an average similarity of 95.4%. The analysis of dataset I indicate M5313 clustering in group A (S1 Fig), with approximately 99% similarity with the two M4147 sequences. Group B, composed of viral strains isolated in French Guiana alone, presented an intra-group average of 97.7% similarity. Group D only included LRV1 sequences from strains of *L*. *guyanensis* from Brazil (from Rondônia, Pará and Amazonas) and presented the lowest intragroup similarity of 86.6% between sequences 353 and 2001. Sequences 3542 and 1398 were only included in Dataset I and clustered in Group D (S1 Fig), with similarities ranging from 98% to 99% for sequence IDs 3503 and 2001. The only sequence of LRV1 from *L*. *shawi* (1545 –MG202142) exhibited the greatest similarity to *L*. *guyanensis* LRV1 sequences in Groups A and B, with the highest similarity (91.3%) to XK73 (Group A) and the lowest to 2001 (Group D). The percentage similarity between each sequence pair is listed in S1 Table.

In addition to the previously reported LRV1 sequences from *L*. *braziliensis* (YA70—KY757610 and Lb2169—KC862308), the other sequences from the strains of the same *Leishmania* species from Bolivia and RO/Brazil were placed in Group F. No identical sequences were identified among *L*. *braziliensis* viruses. Sequence 2169 was analyzed in Dataset I and clustered close to LEM2700, LEM3874, and LEM2780a (S1 Fig) with similarities ranging from 88.1% to 91.3%. The sequences with the highest similarity were 3569 –MG202145 and 3567 –MG202139, both from Rondônia, and the lowest similarity was observed between sequences 3545 –MG202143 and Lb2169 –KC862308, from Rondônia and Bolivia, respectively. LRV1 sequences from *L*. *braziliensis* exhibited 89.7% similarity. Within Group F, the only LRV1 from the *L*. *braziliensis* strain obtained from French Guiana (YA70—KY757610) exhibited between 87.5% and 89.5% similarity to the LRV1 from *L*. *braziliensis* strains from RO and from Bolivia (S1 Table). Although the level of similarity observed among the sequences of Group F (*L*. *braziliensis*) is similar to the inter-group similarity observed for Groups A and B (both consisting of LRV1 from *L*. *guyanensis)*, the results presented in the MinSpanning Network indicate that the *L*. *braziliensis* LRV1 sequences analyzed here form a single group (Fig 4).

## Discussion

We report a total of 13 LRV1 sequences from three different strains of *Leishmania* species circulating throughout Brazil, including the first report in the literature on the virus found in *L*. *shawi*. The region of the LRV1 genome analyzed here is partially composed of *orf*1 and 2; the function of *orf*1 has not yet been defined, and *orf*2 is responsible for encoding the viral capsid protein (CP) [14]. After alignment with other sequences available in GenBank, the analyses performed with fragments of almost 800 base pairs demonstrated the ability to form species-specific clusters, as well as a geographic separation when virus sequences from the same *Leishmania* species were analyzed, such as the formation of *L*. *guyanensis* LRV1 groups and the *L*. *braziliensis* virus group. These results reinforce the endosymbiotic relationship between the virus and the parasite as well as the hypothesis of their coevolution [26].

The methodology presented in the current study suggests that this region may be used for future studies that evaluate the endosymbiotic relationship between LRV1 and different *Leishmania* species. In addition to facilitating such experiments, the results were satisfactory when compared to a recent study that analyzed the complete genome of 24 French Guiana LRV1 sequences and concomitantly analyzed a 299 base pair region of the *orf*2 viral genome including viral sequences from Bolivia and Peru [23]. Our results, which analyze part of the viral genome and add new LRV1 sequences, produce the same groups as the previous study, indicating that the region studied here is phylogenetically informative. These results agree more strongly with those obtained through the analysis of the complete genome than with those obtained when the same authors analyzed a partial sequence of 299 nucleotides from the viral capsid, where Groups A and B of *L*. *guyanensis* were not clearly defined.

In this study, Groups A, B, C, and E maintained the same composition as reported previously [23]. Groups A and B correspond to previously defined groups containing the LRV1 sequences of *L*. *guyanensis* strains from French Guiana [23]. Note that most of the LRV1 sequences from *L*. *guyanensis* from French Guiana are in these groups. Group A, in addition to nine sequences from French Guiana, contained three *L*. *guyanensis* viral sequences from Monte Dourado (Pará State) in Brazil (M5313 –JX313126 and M4147 –UO1899 and KX808487), a city bordering the state of Amapá, which borders French Guiana. Two of these, U01899 and KX808487, came from the same strain of the parasite (M4147) obtained in the years 1994 and 2017, respectively; these sequences exhibited 3 differences in nucleotide position. The observed differences between the two LRV1 sequences of *L*. *guyanensis* strain M4147 may have occurred due to a variety of factors, including *in vitro* parasite maintenance for distinct periods of time and under specific conditions. LRV1 sequencing has been performed by different groups over time [5,28], with differences in sequencing methods and crude sequence analyses potentially leading to some differences. Group B, composed of 11 sequences from *L*. *guyanensis*, showed the lowest divergence between sequences compared to the other groups, and the composition remained similar to that reported by Tirera et al. in 2017.

The new LRV1 sequences from *L*. *guyanensis* presented in this study were all classified into Group D. This group contained a sequence that was previously reported from a strain isolated from a patient who contracted the disease in Manaus, Amazonas. We highlight that in addition to the LRV1 sequences of *L*. *guyanensis* from Amazonas, this group included LRV1 sequences from *L*. *guyanensis* from RO and from PA (3354 –MG202141), although from the city of Santarém. Santarém and Monte Dourado are separated geographically by the Amazon River, an important geographical barrier for vectors, which may lead to the divergence of sequences of this parasitic species.

The phylogenetic tree and the NeighborNet (Figs 2 and 3) show that group D is well-supported and segregated into two well-supported monophyletic clades, similar to the results for Groups A and B. One group is composed of three sequences, two from Amazonas (Manaus) and one from Rondônia (Candeias). The second group is composed of six strains, five from Amazonas (Manaus) and one from the Para state (Santarem). More LRV1 sequences from *L*. *guyanensis* from different Brazilian localities are needed to determine whether group D is split into different geographically linked clusters.

Previous studies with monoclonal antibodies had already demonstrated differences in the reactivity profile between *L*. *guyanensis* isolates from regions in Pará, north of the Amazon River, and from French Guiana in relation to parasites isolated from some regions of the Amazon, including Manaus [38,39]. In fact, when we observed the formation of groups from the sequences analyzed here, we concluded that they converge with studies of parasites using monoclonal antibodies. Groups A and B comprise all viral sequences of the isolates from French Guiana and Pará, whereas in Group D, isolates from Amazonas (Manaus), Pará (Santarém), and a single isolate from Rondônia (Candeias) are observed. Multilocus analyses, either by microsatellite markers (MLMT) or by analysis of nucleotide sequences of various genes (MLSA), show genetic homogeneity in *L*. *guyanensis*. However, based on MLMT, the strains of *L*. *guyanensis* from Manaus form a population, whereas one strain from Acre (the state neighboring RO) remained in a different population, along with other species from the subgenus *Viannia* [40]. MLSA studies showed the formation of a group composed of Sequence Types (ST) of *L*. *guyanensis* from Manaus, excluding two STs from Pará [41]. All the aspects mentioned above reinforce the LRV1-*Leishmania* co-evolution hypothesis.

Observation of the formation of two LRV1 groups from *L*. *guyanensis* was enhanced with the results of MinSpanning Network and NeighborNet, where one of the clusters formed is solely composed of LRV1 sequences from Group D, which includes samples from RO and AM, and one large *L*. *guyanensis* LRV1 cluster composed of all the LRV1 sequences from French Guiana, including the only sequence from Suriname, samples from PA and the single sequence from *L*. *shawi*, also from PA. Several studies point to genetic similarity between *L*. *guyanensis* and *L*. *shawi*, with the latter representing a distinct clonal complex within the *L*. *guyanensis* group [41]. The LRV1 sequence from *L*. *shawi* strain 1545 showed greater similarity to the sequences of Groups A and B. This strain was isolated in Paraupebas, Pará, Brazil, where the circulation of *L*. *guyanensis* strains carrying the B19 epitope is observed [38] in *Leishmania* strains from French Guiana.

Groups C and E both included one LRV1 sequence of L. guyanensis from French Guiana, as previously reported [23]. Group C was more closely related to Groups A and B, and Group E, which also included a sequence from Suriname, was more closely related to Group D. Of note, Group E presented large connection to Groups A, B, and C in the MinSpanning Network. As data on the geographic location of French Guiana and Suriname isolates are not available, it is not possible to infer the extent to which geographic regions drive cluster formation, but this factor could be related to the observed proximity between these groups.

There are three new LRV1 *L*. *guyanensis* sequence public available that were not included in this study. M6200—KX686068 from Brazil clustered in Group A, and two others (KU724433 and KU724434) clustered in Group D (S1 Fig), but we did not incorporate these sequences in the analysis, as it was not possible to trace their geographic origins. This information could contribute to understanding the relationship between LRV1 clustering and geographic dispersion. Based on our results, we hypothesize that M6200 is from Monte Dourado (Pará) or another nearby region, while the other two strains are from localities close to those where we detected LRV1 Group D.

Group F was formed by LRV1 sequences from *L*. *braziliensis*, the group presenting the greatest divergence among its sequences (87.5% to 98.3% of intra-group similarities), confirming reports of heterogeneity for this species [40–43]. Based on the results observed in the phylogenetic tree, there is a tendency for the sequences to cluster according to their geographic origin (Brazil, Bolivia, and French Guyana), but with a low bootstrap value, thus making this a single group denominated Group F. The results of the MinSpanning Network and NeighborNet reinforce the observations from the phylogenetic tree. The MinSpanning Network includes a cluster formed solely by *L*. *braziliensis* sequences, and NeighborNet reveals splits separating the sequences of each country.

The results presented here are based on a small sample number and few geographical regions. A sample of LRV1 sequences with greater geographic distribution is required to confirm the hypothesis of LRV1 and *Leishmania* (*Viannia*) species and population coevolution. Several studies have examined the presence of LRV1 in *Leishmania* strains, and it is noteworthy that the viral endosymbiont has not been detected in regions that likely have the same population of *L*. *braziliensis* as Rio de Janeiro and Minas Gerais [20,24], according to MLMT analyses [40], while the endosymbiont is present in the populations that circulate in the Northern part of South America, including the populations of *L*. *guyanensis*, *L*. *braziliensis* and *L*. *shawi*. It is important to mention that *Leishmania* with and without LRV1 are present in the same region; similarly, in the same culture we can observe cells with and without viral particles [19]. It is possible that a bottleneck phenomenon occurred during the dispersal process of *L*. *braziliensis*, considering that the other species of *L*. (*Viannia*) seem to be restricted to the Amazon region and that parasites without viral particles have been better adapted to the conditions encountered, especially in relation to the phlebotomine species.

## Conclusions

Population mixing is likely to be an important determinant of coevolution [44]. As previously demonstrated, LRV1 is transmitted intracellularly during cell division. Mixed infections of the same *Leishmania* culture with two LRV1 strains has already been observed [23], as well as *Leishmania* cells infected and non-infected by LRV1 in the same culture [19].

Recently, it was suggested that the *Leishmania* RNA virus infected *Leishmania* parasites at the same time that the dixenous life cycle appeared in Leishmaniinae [25] and that the virus coevolved with these parasites since that time, which was prior to the divergence of *Leishmania* subgenera. The mechanisms that determine *Leishmania*-*Leishmania* RNA Virus specificity are not documented or understood as they are for other groups of parasites. The consequences of *Leishmania*-*Leishmania* RNA Virus coevolution are reliant on co-evolutionary dynamics and likely involve both fluctuating selection and antagonist coevolution (directional towards increasing infectivity/resistance). Under fluctuating selection, there are oscillations in the frequency of genotypes with particular resistance and infectivity [45] or fluctuations in the range of resistance and infectivity [46]. *Leishmania* species and populations are spatially structured [40,41], and the extent of gene flow between populations can alter co-evolutionary dynamics and result in patterns of local adaptation. This explains the fact that the dispersion of *L*. *braziliensis* populations without LRV1, which may represent a *Leishmania* population resistant to the virus, stabilized after the process of coevolution. There is an important entanglement of ecology and evolution in the course of *Leishmania*-LRV1 coevolution. The interaction of *Leishmania* species with their hosts had a direct impact on this evolutionary process.

The analyses presented here corroborated the group assignment of previous studies based on the complete genome sequences of LRV1 [23]. For this reason, along with the ability to access shorter sequence regions, we suggest applying this approach to future studies on the molecular systematics of LRV1.

## Supporting information

S1 Table Nucleotide identities (%) between LRV1 sequences. (PDF)

S1 Fig Molecular phylogenetic analysis by maximum likelihood method involving 50 LRV1 nucleotide sequences. (TIF)
