# Supplementary figures and images for: New insights into the genetic diversity of Leishmania RNA Virus 1 and its species-specific relationship with Leishmania parasites
Source: PLoS One. 2018 Jun 18;13(6):e0198727. doi: 10.1371/journal.pone.0198727 (PMC6005476; doi:10.1371/journal.pone.0198727)

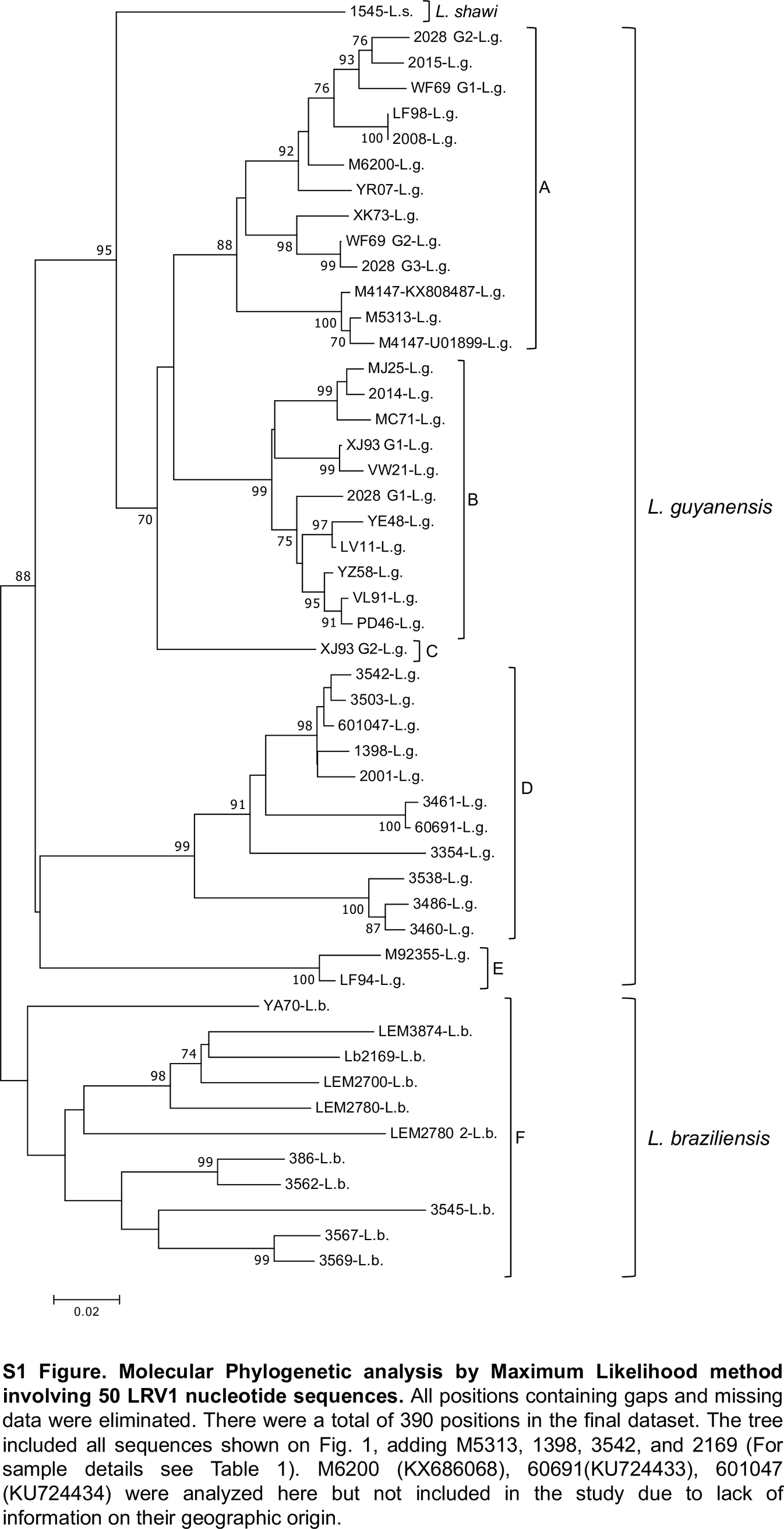

Supplement: S1 Fig — (TIF) [file pone.0198727.s002.tif]
