# Supplementary material for: New insights into the genetic diversity of Leishmania RNA Virus 1 and its species-specific relationship with Leishmania parasites
Source: PLoS One. 2018 Jun 18;13(6):e0198727. doi: 10.1371/journal.pone.0198727 (PMC6005476; doi:10.1371/journal.pone.0198727)
Supplement: S1 Table — (PDF) [file pone.0198727.s001.pdf]

S1 Table - Nucleotide identities (%) between LRV1 sequences

Groups		F (L.b.)										A (L.g.)											B (L.g.)											C (L.g.)	D (L.g.)							E (L.g.)		L.s.
		3569	386	3562	YA70	LEM2700	LEM2780(a)	3567	LEM2780(b)	3545	LEM3874	2008	WF69(G1)	2015	LF98	2028(G3)	2028(G2)	WF69(G2)	XK73	YR07	M4147 (KX808487)	M4147 (U01899)	PD46	YZ58	VL91	XJ93	VW21	YE48	MJ25	2028(G1)	2014	MC71	LL28	XJ93(G2)	3460	3461	3538	3354	2001	3503	3486	LF94	CUMC1	1545
F (L.b.)	3569	-																																										
	386	94.5																																										
	3562	93.2	97.2																																									
	YA70	89.2	89.8	89.8																																								
	LEM2700	91.4	89.1	89.7	88.8																																							
	LEM2780(a)	90.2	89.4	89.2	87.6	94.0																																						
	3567	98.3	93.9	93.2	88.9	90.8	89.7																																					
	LEM2780(b)	89.7	87.9	88.1	87.5	90.5	91.1	89.8																																				
	3545	92.3	91.1	91.4	89.5	88.4	88.2	91.7	87.8																																			
	LEM3874	89.8	88.5	88.1	88.8	94.3	92.7	89.5	89.2	88.2																																		
A (L.g.)	2008	86.9	87.8	87.2	85.7	86.3	86.3	86.5	85.6	84.0	86.0																																	
	WF69(G1)	86.0	87.2	86.8	85.3	86.8	86.2	85.7	84.7	84.1	86.3	97.8																																
	2015	85.6	86.6	86.2	84.7	86.0	85.4	85.6	84.9	83.6	85.6	97.4	98.3																															
	LF98	86.8	87.6	87.0	85.9	86.5	86.3	86.3	85.4	83.8	86.0	99.9	97.8	97.4																														
	2028(G3)	86.5	86.5	86.9	86.2	86.6	85.7	85.9	85.4	84.4	85.3	94.8	94.2	94.3	94.8																													
	2028(G2)	85.6	86.9	86.5	85.2	86.0	85.4	85.3	84.4	83.7	85.6	97.5	98.1	98.7	97.5	94.2																												
	WF69(G2)	86.6	86.6	87.0	86.3	86.8	85.9	86.2	85.4	84.4	85.4	94.9	94.3	94.5	94.9	99.6	94.3																											
	XK73	86.3	86.8	87.2	85.6	86.6	85.2	86.0	85.6	84.6	84.9	94.8	94.5	94.5	94.8	97.4	94.2	97.5																										
	YR07	86.5	87.3	87.0	86.5	86.8	86.2	86.3	85.6	84.0	85.9	97.7	96.9	96.8	97.7	96.2	96.9	96.1	95.3																									
	M4147 (KX808487)	85.9	85.9	86.2	85.4	85.6	85.2	85.7	84.9	83.1	85.2	95.5	95.1	94.6	95.5	95.1	94.8	95.1	94.6	95.3																								
	M4147 (U01899)	85.6	85.6	85.9	85.2	85.3	84.9	85.4	84.6	82.8	85.0	95.2	94.8	94.3	95.2	94.9	94.5	94.9	94.3	95.1	99.6																							
B (L.g.)	PD46	86.8	86.5	86.2	86.6	86.5	86.3	86.2	85.2	84.6	86.5	92.3	92.4	92.1	92.3	93.0	92.4	93.2	93.2	93.0	92.7	92.6																						
	YZ58	86.9	86.9	86.8	87.0	86.6	86.5	86.3	85.3	84.9	86.6	92.9	93.0	92.4	92.9	93.6	92.7	93.7	93.7	93.6	93.3	93.2	99.4																					
	VL91	86.8	86.8	86.5	86.9	86.8	86.6	86.2	85.4	84.9	86.8	92.6	92.7	92.4	92.6	93.2	92.7	93.3	93.4	93.2	92.7	92.6	99.7	99.4																				
	XJ93	85.4	86.2	86.2	86.5	85.4	86.3	84.9	84.6	84.6	86.6	92.3	91.8	92.0	92.3	93.2	92.0	93.3	93.2	92.7	92.6	92.4	97.2	97.5	97.2																			
	VW21	85.6	86.3	86.3	86.3	86.0	86.6	85.0	85.0	84.7	87.2	92.3	91.8	92.0	92.3	93.2	92.0	93.3	93.2	93.0	92.6	92.4	96.7	96.9	96.7	99.4																		
	YE48	87.0	87.0	87.3	87.2	87.0	86.3	86.5	85.3	84.9	86.6	92.1	92.1	91.8	92.1	93.0	92.1	93.2	92.9	93.0	92.6	92.4	98.1	98.4	98.1	97.1	96.8																	
	MJ25	86.3	86.8	86.8	87.0	86.2	86.8	85.7	85.3	84.9	86.6	92.4	92.1	91.8	92.4	92.9	92.1	93.0	92.9	92.4	92.1	92.0	97.1	97.4	97.4	97.5	96.9	96.9																
	2028(G1)	86.2	86.8	86.8	86.8	85.9	85.7	85.9	85.2	84.7	86.0	92.1	92.1	92.0	92.1	92.9	92.3	93.0	93.0	92.9	92.7	92.6	98.3	98.5	98.3	97.7	97.1	98.1	97.1															
	2014	85.9	86.6	86.6	87.5	85.6	86.3	85.3	84.9	84.9	86.5	92.3	92.0	91.7	92.3	92.7	92.0	92.9	92.7	92.4	92.0	91.8	97.1	97.4	97.4	97.5	96.9	96.9	99.4	97.4														
	MC71	86.3	86.5	86.5	87.2	86.2	86.3	85.7	85.3	84.9	86.2	92.0	91.7	91.7	92.0	92.7	92.0	92.9	92.7	92.4	92.0	91.8	97.1	97.4	97.4	96.9	96.4	97.1	99.1	97.1	98.8													
	LL28	86.6	86.6	86.9	86.9	86.6	86.2	86.0	85.2	84.6	86.2	92.6	92.7	92.4	92.6	93.3	92.7	93.4	93.2	93.3	93.2	93.0	98.7	99.0	98.7	97.5	96.9	99.4	97.2	98.7	97.2	97.2												
C (L.g.)	XJ93(G2)	86.5	86.2	86.3	87.0	87.3	86.6	86.0	85.6	84.9	87.2	91.1	91.0	90.2	91.0	92.1	90.5	92.0	91.4	91.7	91.7	91.4	93.0	93.4	93.3	93.0	93.3	92.7	93.3	93.4	93.3	93.0	93.3											
D (L.g.)	3460	85.6	84.9	84.4	86.2	87.3	86.3	85.3	85.7	84.4	86.2	86.2	86.6	86.3	86.3	87.2	85.9	87.0	86.5	87.5	85.3	85.0	86.6	86.6	86.5	85.4	85.9	86.9	85.9	86.5	85.9	85.6	86.6	86.3										
	3461	84.7	84.6	83.6	85.2	86.6	87.3	84.9	84.4	84.4	85.9	86.9	86.3	86.2	86.9	86.3	86.0	86.5	86.6	87.0	86.2	85.9	85.9	86.0	85.7	85.9	86.0	85.9	86.0	85.7	86.0	85.4	86.2	86.5	92.1									
	3538	85.4	84.7	84.1	85.7	87.0	86.6	85.2	86.0	84.0	85.7	85.9	86.6	86.3	85.9	87.2	85.9	87.0	86.5	87.5	85.6	85.3	86.9	86.9	86.8	85.7	86.2	86.9	85.9	86.8	85.9	85.6	86.9	86.5	98.4	92.3								
	3354	84.1	84.1	83.3	84.0	86.5	85.3	84.0	83.1	83.0	84.4	87.2	87.0	86.8	87.2	87.0	86.3	87.2	87.3	87.3	87.0	86.8	86.6	86.8	86.5	86.2	86.3	86.5	86.3	86.2	86.5	86.3	86.5	86.8	91.4	94.2	91.6							
	2001	80.9	80.1	79.6	79.9	81.7	82.2	80.8	80.3	80.3	81.1	82.0	81.1	81.4	82.0	82.1	80.9	82.2	82.0	82.4	80.9	80.6	82.4	82.2	82.2	82.5	82.7	82.0	82.5	81.8	82.2	82.1	81.8	82.1	86.8	89.8	86.6	88.9						
	3503	85.9	84.7	84.0	85.3	87.0	87.8	85.7	85.7	84.7	86.6	87.8	86.9	87.0	87.8	87.8	86.6	87.9	87.2	88.1	86.5	86.2	87.9	87.8	87.8	87.8	87.9	87.5	87.8	87.2	87.8	87.2	87.3	87.6	93.0	95.5	92.6	94.8	93.3					
	3486	85.2	84.3	83.8	85.4	86.8	86.3	84.9	85.6	84.0	85.9	86.0	86.6	86.3	86.0	87.5	85.9	87.3	86.8	87.6	85.6	85.3	86.9	86.9	86.8	86.0	86.5	86.9	85.9	86.8	85.9	85.6	86.9	86.8	99.1	92.3	98.4	91.7	87.0	93.3				
E (L.g.)	LF94	86.9	86.9	86.2	85.3	86.6	86.9	85.6	85.6	85.4	86.8	88.5	88.4	87.8	88.5	89.4	87.5	89.2	89.1	88.6	87.8	87.5	87.3	87.8	87.6	87.3	87.6	87.9	87.9	87.8	87.5	87.3	87.6	88.2	87.0	87.3	87.8	87.5	82.1	87.3	87.2			
	CUMC1	86.5	86.2	85.4	85.2	86.2	86.2	85.4	85.2	84.9	86.3	87.5	87.8	86.6	87.5	88.4	86.6	88.2	87.9	87.6	86.8	86.5	86.6	87.0	86.9	86.9	87.2	87.2	87.5	87.0	87.0	86.9	86.9	88.4	86.6	86.3	87.3	87.0	81.4	86.6	87.0	98.4		
L.s.	1545	85.9	86.5	86.3	85.6	86.6	85.6	86.2	84.9	83.6	84.9	89.8	90.2	90.2	89.8	90.8	89.7	91.1	91.3	90.0	90.4	90.2	90.4	90.7	90.7	89.8	90.0	89.7	90.8	90.2	90.5	90.0	90.0	89.7	86.0	85.4	86.0	85.9	80.6	86.5	86.5	86.5	86.2	-

Groups A to F according to Tirera et al., 2017. L.b. = *Leishmania braziliensis; L.g. = Leishmania guyanensis; L.s.= Leishmania shawi. Intra groups similarities are indicated by collors: yellow Group F; blue Group A; pink Group B; grey Group D and green Group E.*
